# Baby Shampoo to Relieve the Discomfort of Tear Gas and Pepper Spray Exposure: A Randomized Controlled Trial

**DOI:** 10.5811/westjem.2017.12.36307

**Published:** 2018-02-26

**Authors:** Jason P. Stopyra, James E. Winslow, James C. Johnson, Keith D. Hill, William P. Bozeman

**Affiliations:** Wake Forest University School of Medicine, Department of Emergency Medicine, Winston-Salem, North Carolina

## Abstract

**Introduction:**

Oleoresin capsicum (OC) or pepper spray, and tear gas (CS) are used by police and the military and produce severe discomfort. Some have proposed that washing with baby shampoo helps reduce this discomfort.

**Methods:**

We conducted a prospective, randomized, controlled study to determine if baby shampoo is effective in reducing the severity and duration of these effects. Study subjects included volunteers undergoing OC or CS exposure as part of their police or military training. After standardized exposure to OC or CS all subjects were allowed to irrigate their eyes and skin ad lib with water. Those randomized to the intervention group were provided with baby shampoo for application to their head, neck, and face. Participants rated their subjective discomfort in two domains on a scale of 0–10 at 0, 3, 5, 10, and 15 minutes. We performed statistical analysis using a two-tailed Mann-Whitney Test.

**Results:**

There were 58 participants. Of 40 subjects in the OC arm of the study, there were no significant differences in the ocular or respiratory discomfort at any of the time points between control (n=19) and intervention (n=21) groups. Of 18 subjects in the CS arm, there were no significant differences in the ocular or skin discomfort at any of the time points between control (n=8) and intervention (n=10) groups.

**Conclusion:**

Irrigation with water and baby shampoo provides no better relief from OC- or CS-induced discomfort than irrigation with water alone.

## INTRODUCTION

### Background

Chemical irritant agents are sometimes used by law enforcement officers and by the military to subdue violent or threatening subjects and to control crowds.^1^,^2^ The most common of these agents include oleoresin capsicum (OC), commonly known as pepper spray, and ortho-chlorobenzylidene malononitrile (CS), commonly known as tear gas. Both cause pain and irritation of the eyes, skin and mucosal surfaces.^3^ Aerosolized OC preparations are also available to the general public in the United States as a self-defense weapon.

### Importance

Pain and irritation from these agents last 15 minutes or more.^3^ While many recommendations exist for decontamination and treatment of discomfort produced by these irritants, the effectiveness of these methods have not yet been demonstrated. Two of the common methods used for decontamination are irrigation with tap water and blowing cool air onto the face and eyes.^3^ Some advocate the use of baby shampoo combined with water irrigation; it is theorized that shampoo can emulsify and enhance removal of the irritant molecules and may reduce nociceptor stimulation, thus reducing the severity and duration of irritant effects.^4^ Medical personnel in the prehospital and emergency department (ED) settings, police and military personnel, as well as individuals exposed to these chemicals, would benefit from evidence-based recommendations for effective decontamination and treatment to reduce the severity and duration of discomfort caused by these agents.

### Goal of This Investigation

We performed a prospective, randomized, controlled trial to determine whether irrigation with water and baby shampoo was superior to water irrigation alone in relieving the acute symptoms produced by tear gas and pepper spray.

## METHODS

### Study Design, Setting and Selection of Participants

We performed a prospective, randomized, controlled study. Volunteer study subjects were police recruits who underwent OC (pepper spray) exposure as part of their training and U.S. Army soldiers who underwent CS (tear gas) exposure as part of their training. The study was approved by the institutional review board. We obtained written, informed consent from each participant prior to study participation. This work was not pre-registered as a clinical trial because the study population’s noxious intervention was part of their externally required training.

### Interventions

As part of their standard training, each participant received a standardized irritant exposure and completed a training evolution. Police recruits received a two-second (approximate) burst of police issue OC spray (First Defense MK-3, Safariland / Defense Technology, Jacksonville, FL) to the face. They were then required to complete a series of tasks to simulate control and apprehension of a combative criminal suspect. This training sequence lasted approximately 1½-2 minutes.

Military trainees wearing protective gas masks were placed in an enclosed structure that was then saturated with CS gas (No. 98 CS grenade, Smith & Wesson / Lake Erie Chemical Company, Wickliffe, OH). Gas masks were removed and each trainee was exposed to the tear gas for approximately 10 seconds. They were then required to perform a series of training tasks and safely exit the multi-story structure. This training sequence also lasted approximately 1½-2 minutes.

After irritant exposure and completion of their training sequence, all subjects proceeded to a decontamination area and were allowed to irrigate their eyes and skin ad lib with water. Participants were randomized to a control group (water irrigation alone) and intervention group (baby shampoo plus water irrigation). The intervention group was provided a cup containing a unit “dose” of 15cc of Johnson’s® baby shampoo (Johnson & Johnson, New Brunswick, NJ) and instructed to apply it liberally to their head, neck, and face. Repeat shampoo “doses” were available ad lib to this group.

**Population Health Research Capsule**

What do we already know about this issue?

Chemical irritant agents used by law enforcement officers to subdue threatening subjects do not have an evidenced-based recommendation for effective decontamination.

What was the research question?

Is water plus baby shampoo or water irrigation alone superior in relieving the acute symptoms produced by tear gas and pepper spray?

What was the major finding of the study?

Irrigation with water plus baby shampoo provides no better relief from pepper spray- or tear gas-induced discomfort than with water alone.

How does this improve population health?

Similar investigations of proposed decontamination agents should be performed to provide evidence of their efficacy prior to their adoption and deployment.

**Figure 1:**
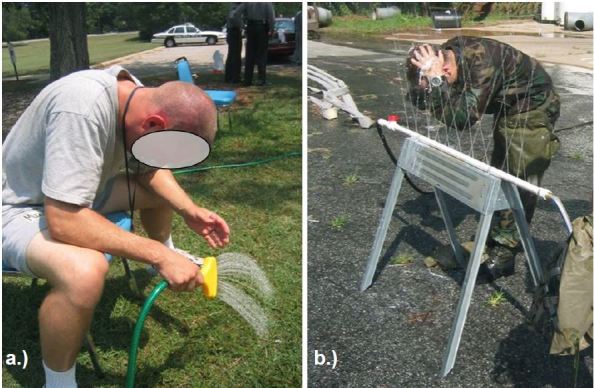
Irrigation technique and equipment for a) OC (pepper spray), and b) CS (tear gas).

Irrigation was provided by a garden hose for police trainees exposed to OC and by a custom-made, multi-station irrigation device for military trainees exposed to CS. This device was constructed of two PVC pipes supported horizontally three feet off the ground and connected to a fire hydrant. Water flow was adjusted to produce an approximately 48-inch column of water from each of 20 holes drilled in each PVC pipe at offset angles (Figure 1).

### Outcomes

Subjects verbally rated their discomfort in two domains using a Likert scale of 0 to 10, with zero indicating no discomfort and 10 indicating maximal discomfort. Assessments were recorded at 0, 3, 5, 10, and 15 minutes, with timing starting upon entry to the decontamination area and the first assessment performed prior to any decontamination efforts. Eye and respiratory discomfort were assessed after OC exposure, and eye and skin discomfort were assessed after CS exposure, based on previous experience of the most prominent areas of discomfort with each agent. Per usual training practice, once subjects were subjectively recovered to a sufficient degree to comfortably converse and ambulate, the decontamination segment was concluded and they were allowed to exit the decontamination area to continue with their training activities. There were no protocol violations.

### Power Calculation

We performed a power calculation presuming a 95% confidence to detect a clinically significant mean difference of two points on a 10-point Likert scale of discomfort, a standard deviation of one among participants rating their discomfort, alpha = 0.05, and a two-tailed t-test. This revealed that at least eight pairs of subjects (16 subjects total) were required in each of the two exposure groups. (StatMate version 2.00 for Windows, GraphPad Software, San Diego, CA)

### Analysis

We entered data into a spreadsheet (Microsoft Excel, Redmond, WA) and performed descriptive analysis. Statistical software (InStat version 3.10 for Windows, GraphPad Software, San Diego, CA) was used to compare groups using the nonparametric Mann-Whitney test to compare means at each time period. We considered a p-value of ≤0.05 statistically significant.

## RESULTS

### OC (Pepper Spray) Exposure arm

Forty law enforcement recruits received OC exposure and completed the study protocol. The mean age was 28 years (range 21 to 38); 39 of the participants (98%) were male.

**Figure 2:**
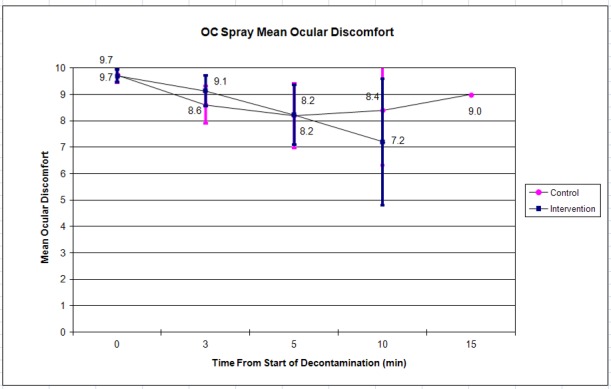
Mean ocular discomfort after OC (pepper spray) exposure. Error bars indicate 95% confidence interval.

**Figure 3:**
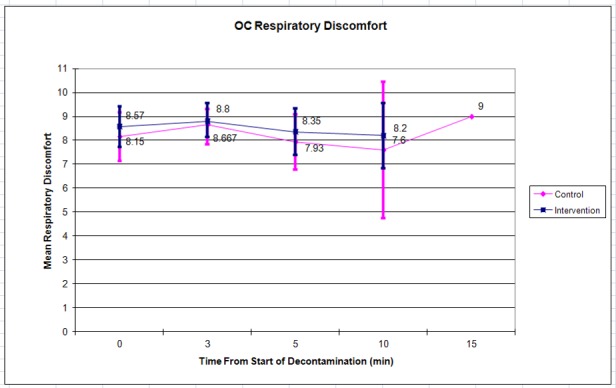
Mean respiratory discomfort after OC (pepper spray) exposure. Error bars indicate 95% confidence interval.

The control (n =19) and intervention (n =21) groups reported similar mean initial ocular discomfort of 9.6 vs. 9.7 respectively. This decreased to 8.7 vs. 7.2 by 10 minutes (Figure 2). Mean respiratory discomfort was initially 8.2 vs. 8.6, and changed only slightly to 9.0 vs. 8.2 by 10 minutes (Figure 3). There were no statistically significant differences between the control and intervention groups at any of the time points.

Standard deviation of discomfort ratings after OC exposure averaged 1.66. Participant attrition was significant after 3–5 minutes, as subjects felt improved and left the decontamination area per standard training practice. This resulted in 39 subjects contributing data at the 0 and 3-minute marks, 32 at the 5-minute mark, 10 at the 10-minute mark, and one at the 15-minute mark.

### CS (Tear Gas) Exposure arm

Eighteen soldiers received CS exposure. The mean age was 26 years (range 20 to 36); 17 of the participants (94%) were male.

**Figure 4:**
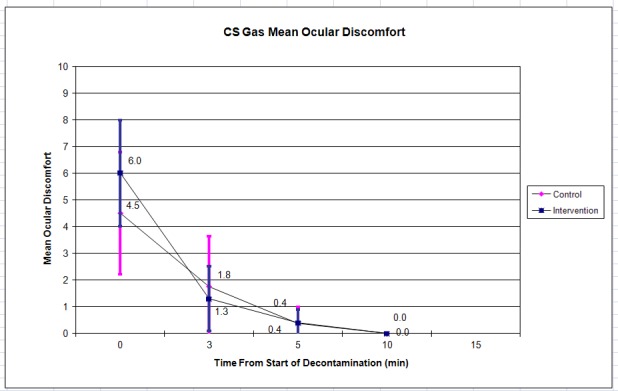
Mean ocular discomfort after CS (tear gas) gas exposure. Error bars indicate 95% confidence interval.

**Figure 5:**
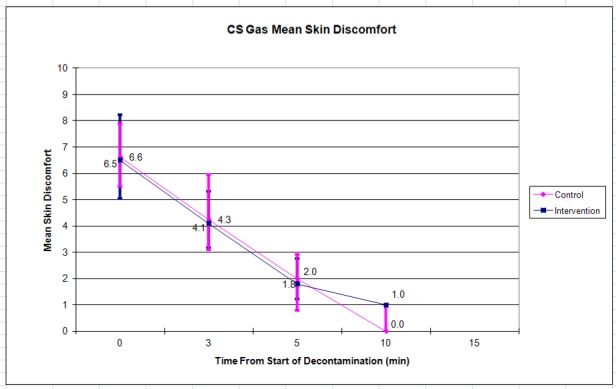
Mean skin discomfort after CS (tear gas) gas exposure. Error bars indicate 95% confidence interval.

The control (n = 8) and intervention (n = 10) groups had mean initial ocular discomfort of 4.5 vs. 6.0. This decreased to zero for both groups at 10 minutes (Figure 4). Mean initial skin discomfort was 6.6 vs. 6.5, and declined to 0.0 vs. 1.0 by 10 minutes (Figure 5). There were no statistically significant differences between the control and intervention groups at any of the time points.

Standard deviation of discomfort ratings after CS exposure averaged 1.75. Participant attrition was also significant in this study arm as subjects felt improved and left the decontamination area per standard training practice. This resulted in 18 subjects contributing data at the 0, 3- and 5=minute marks, four at the 10-minute mark, and zero at the 15-minute mark.

## DISCUSSION

Oleoresin capsicum (OC) or “pepper spray” is an oil-based extract from pepper plants of the genus *Capsicum.* The chemically active ingredient is capsaicin, a fat-soluble phenol. OC causes its effect by stimulating type C unmyelinated nerve fibers that cause the release of substance P along with other neuropeptides, causing neurogenic inflammation and vasodilation.^5^ These neuropeptides also produce protective reactions of mucus secretion and coughing.^6^ Clinically this results in a painful burning sensation of the skin and mucous membranes, blepharospasm (involuntary closing of the eyes), and shortness of breath. Although OC causes a prominent subjective sense of dyspnea due to mucosal irritation, research has shown no objective change in respiratory function.^7^ OC has been estimated to be 90% effective in stopping aggressive behavior.^6^ A prior review of ED visits for OC exposure found the most common symptoms to be burning, erythema and local irritation to exposed areas.^8^

“Tear gas” is a lay term used to describe a group of irritant chemicals that cause lacrimation. The most commonly used agent by law enforcement is CS. CS is actually a crystalline solid, not a gas, making the term “tear gas” a misnomer; it is insoluble in water and has a small solubility in alcohols.^3^ It is aerosolized by multiple techniques including dissolving it in an organic solvent, micro-pulverization into a powder or in use with a thermal grenade that produces hot gases. The most notable acute effects of CS are lacrimation, ocular pain, and blepharospasm.^9^ Skin discomfort is also common, and skin erythema and blistering have been seen in rare instances of prolonged exposure.^10^ CS also produces irritant effects on other mucosal surfaces and can produce pulmonary symptoms of subjective dyspnea, coughing, and rarely bronchospasm.^11^

A number of topical decontamination agents have been proposed for use after OC and/or CS exposure, including proprietary mixtures sold commercially for decontamination purposes. Despite advertising claims of efficacy, none of these products to the authors’ knowledge have been demonstrated to be effective in the published literature to date.

The results of this study indicate that the use of baby shampoo as a decontamination agent after OC and CS exposure provides no better or faster relief from acute symptoms than water irrigation alone. These findings are consistent with a previous study of several potential decontamination agents for OC exposure, including Maalox®, 2% lidocaine gel, milk, and baby shampoo, compared to water irrigation alone.^12^ That small but well-done, prospective, randomized study included 10 subjects in each group and did not suggest efficacy for any of the agents studied. The present study confirms and expands on that work by adding a larger group of 40 OC-exposed subjects with serial post-exposure symptom assessments and by extending the evaluation to 18 subjects exposed to CS as well.

A second previous study examined a limited dermal exposure of OC to both forearms of 10 volunteers. Those researchers found that a Maalox®-impregnated, topical dressing provided a small amount of pain relief compared to a saline-impregnated dressing.^13^ As acknowledged by the authors, their small study has limited clinical application, as it did not include ocular or mucosal exposure to OC, as typically occurs in law enforcement or self-defense uses.

Both pepper spray and tear gas are known to be generally safe and effective in modifying the behavior of criminal suspects and crowds.^1^,^2^ Their use will likely remain common for the foreseeable future in law enforcement and self-defense settings, and for crowd control. Although these agents rarely produce significant injuries, illness, or fatalities, they are profoundly uncomfortable. Medical and public safety personnel must remain diligent in screening and identifying the minority of exposed subjects who do suffer medical complications, and they should be familiar with the effects of these agents and with appropriate post-exposure decontamination and treatment procedures.

## LIMITATIONS

There are several limitations to this investigation. The amount, frequency, and specific method of shampoo application could not be fully standardized in the participants. While a practical and realistic approximation of real-world utilization, it is possible that this intuitive application method did not optimally deliver the shampoo to the eyes or elsewhere and that another method of application could provide additional relief. We assessed only two parameters of discomfort for each type of exposure (eye and respiratory discomfort after OC; eye and skin discomfort after CS). These parameters were chosen based on prior experience and reports, and this was an intentional decision based on concern that assessment of numerous parameters would result in global rather than specific discomfort ratings in subjects experiencing marked acute discomfort.

Subjects were permitted to leave the decontamination area at their own discretion when they felt ready to continue with their training per traditional training practices; most left prior to the 10- or 15-minute assessment point, leaving few data points and wide confidence intervals (as shown in Figures 2–5) at this time point. Future iterations of this study should include mandatory participation throughout the assessment period to counter this. In addition, future studies with similar methods may use the standard deviations in discomfort ratings that we observed among participants (which were larger than anticipated) in calculating needed sample sizes.

Small sample sizes in this study limited the ability to assess small differences in discomfort ratings. It was not possible to blind the investigators or participants to the agent they received, as an inert lathering agent was not available and may have confounded results if used. It was felt that the prospective, randomized, controlled trial design was the best counter to this limitation. Lastly, the possibility of a placebo effect should not be discounted; individuals who use a “special” decontamination agent to address their discomfort may have an expectation that their symptoms, no matter how severe, are less than what they might experience had they not used the decontamination agent. This expectation may result in belief that the agent is effective based on personal (i.e., anecdotal) experience.

## CONCLUSION

This study demonstrates that the addition of baby shampoo to water irrigation does not appear to reduce the severity or duration of the acute discomfort produced by pepper spray or tear gas exposure. Similar investigations of proposed decontamination agents should be performed to provide evidence of their efficacy prior to their adoption and deployment.
